# Remarks on Delirium Tremens

**Published:** 1839-01-26

**Authors:** William H. Klapp

**Affiliations:** Philadelphia


### REMARKS ON DELIRIUM TREMENS.

By William H. Klapp, M. D.

To the Editors of the Medical Examiner.

*Gentlemen:*—In the last number but one of your very valuable periodical, you have reported a lecture of Dr. Coates on delirium tremens; and, in casting my eye over it, I perceive that the Doctor has been betrayed into an error respecting my practice in the Moyamensing Prison. As the friends of truth and medical science, I am persuaded neither yourselves nor the ingenious lecturer can have the slightest objection to have this error corrected. For the better information, then, of the profession, I can assure you that it has not been in "instances of recent drunkenness," that emetics have been so successfully used by me at the prison, but in real, bonâ fide cases of the disease in question, that is, in true delirium tremens, characterized by different morbid perceptions, or, if you please, by "delirium with phantoms." It has, I think, been in other places suggested, as well as in the lecture of my friend Dr. Coates, that the cases in which emetics have been efficacious, were, in reality, instances of drunkenness, and not of the disease under consideration—in other words, that an error in diagnosis had been committed. This version of the subject will, at once, appear very improbable, when the well known experience and intelligence of the different writers are considered, who, in different quarters of the United States, as well as in Europe, have expressed to the profession their approbation of the practice. In reference, however, to the practice in the Moyamensing Prison, I can most confidently assert there has been no mistake upon the subject. The keepers of that institution well know that the cases in question did not immediately arise from drunkenness, but occurred, chiefly, after the lapse of from three days' to a week's incarceration, and entire restriction from alcoholic drinks, which the discipline of the prison does not permit the use of. I therefore trust, that the superiority of the method of cure which I have adopted, and am in the daily use of, cannot, and will not be explained away in any such manner. The practice pursued by me in this disease, is that which Dr. Joseph Klapp, of this city, many years ago, recommended,—the same as that, by means of which, Dr. Ware, of Boston, was lately able to cure *eleven* cases out of twelve; and the twelfth, in all probability, would have yielded to the treatment, had not the case happened to have been complicated with severe disease of the brain. In conclusion, allow me to state, that in fifty-one cases treated by me in this institution, all but one have been cured *without the use of a grain of opium, or a drop of alcoholic drink of any description.* The unsuccessful case was a female, who died in the night of the day on which she was attacked, of epilepsy, a disease with which she had been previously affected for several years. I have no doubt, the results of this practice with me, will be regarded by some as extraordinary, and it is possible my success has been greater than will again occur in a similar number of cases. But to such as may be disposed to hesitation on the subject, the best thing I can say, is, that the correctness of my report can, at any time, be authenticated by a reference to the case book at the prison, which is accessible to any one who may be disposed to look into it for himself. It is true, we possess in that institution, many advantages as respects treatment. The cases originate under our immediate notice, and, consequently, we are afforded a favourable opportunity of resorting to medical means in their early stages. The disease, too, I think, is more frequently met with in a distinct, uncomplicated state, than in the other institutions of our city, and, of course, more readily susceptible of relief.

I remain, gentlemen,

With great respect, yours, &c.

WILLIAM H. KLAPP.

*Philadelphia*, January 15th, 1839.

